# Nerve growth factor reduces amiloride‐sensitive Na^+^ transport in human airway epithelial cells

**DOI:** 10.14814/phy2.12073

**Published:** 2014-07-17

**Authors:** Michael J. Shimko, Eric J. Zaccone, Janet A. Thompson, Diane Schwegler‐Berry, Michael L. Kashon, Jeffrey S. Fedan

**Affiliations:** 1 Department of Pharmaceutical and Pharmacological Sciences, West Virginia University, Morgantown, West Virginia; 2 Pathology and Physiology Research Branch, NIOSH, Morgantown, West Virginia

**Keywords:** Airway epithelium, electrophysiology, ion transport, lung, nerve growth factor

## Abstract

Nerve growth factor (NGF) is overexpressed in patients with inflammatory lung diseases, including virus infections. Airway surface liquid (ASL), which is regulated by epithelial cell ion transport, is essential for normal lung function. No information is available regarding the effect of NGF on ion transport of airway epithelium. To investigate whether NGF can affect ion transport, human primary air‐interface cultured epithelial cells were placed in Ussing chambers to obtain transepithelial voltage (−7.1 ± 3.4 mV), short‐circuit current (*I*_sc_, 5.9 ± 1.0 *μ*A), and transepithelial resistance (750 *Ω*·cm^2^), and to measure responses to ion transport inhibitors. Amiloride (apical, 3.5 × 10^−5^ mol/L) decreased *I*_sc_ by 55.3%. Apically applied NGF (1 ng/mL) reduced *I*_sc_ by 5.3% in 5 min; basolaterally applied NGF had no effect. The response to amiloride was reduced (41.6%) in the presence of NGF. K‐252a (10 nmol/L, apical) did not itself affect Na^+^ transport, but it attenuated the NGF‐induced reduction in Na^+^ transport, indicating the participation of the trkA receptor in the NGF‐induced reduction in Na^+^ transport. PD‐98059 (30 *μ*mol/L, apical and basolateral) did not itself affect Na^+^ transport, but attenuated the NGF‐induced reduction in Na^+^ transport, indicating that trkA activated the Erk 1/2 signaling cascade. NGF stimulated phosphorylation of Erk 1/2 and the β‐subunit of ENaC. K‐252a and PD‐98059 inhibited these responses. NGF had no effect on *I*_sc_ in the presence of apical nystatin (50 *μ*mol/L). These results indicate that NGF inhibits Na^+^ transport through a trkA‐Erk 1/2‐activated signaling pathway linked to ENaC phosphorylation.

## Abstract (precis)

Nerve growth factor (apical, 1 ng/mL) reduced amiloride‐sensitive Na^+^ transport in human cultured bronchial epithelial cells. We determined that this reduction in Na^+^ transport occurred through NGF‐mediated activation of the trkA receptor and Erk 1/2 signaling cascade to cause phosphorylation of ENaC.

## Introduction

Nerve growth factor (NGF), the first member of the neurotrophin family discovered by Levi‐Montalcini (Cohen et al. 1954), is involved in the development, growth, and survival of sympathetic nerves. Although NGF was discovered in the context of nerve growth and function, it has been shown to be produced by both structural and nonstructural cells in the lung (Hoyle 2003; Frossard et al. 2004). NGF is involved in the development of several airway diseases, such as asthma, and neurogenic inflammation (Braun et al. 1998; Nassenstein et al. 2006). Elevated levels of NGF have been shown to cause airway hyperreactivity, enhance the airway inflammatory response in ovalbumin‐sensitized mice, and cause airway remodeling (Braun et al. 1998; Freund and Frossard 2004). Both NGF and its receptors, trkA and p75, are upregulated during respiratory syncytial virus infections (Hu et al. 2002; Tortorolo et al. 2005). No studies have been conducted to investigate the effect of NGF on ion transport in airway epithelial cells.

In addition to acting as a physical barrier, the polarized airway epithelial cells maintain the airway surface liquid (ASL), which is composed of a periciliary liquid layer (PCL) and a mucus phase. The PCL is necessary for mucociliary clearance of infectious organisms and inhaled particles (Toczylowska‐Maminska and Dolowy 2012). The PCL is maintained by the coordinated action of many ion channels, pumps, and transporters (Knowles et al. 1984; Toczylowska‐Maminska and Dolowy 2012). Disruption of ion transport can contribute to airway diseases, such as mucus thickening in cystic fibrosis due to PCL dehydration, lung edema due to an inhibition of epithelial Na^+^ channels (ENaC; Chen et al. 2004; Morty et al. 2007; Ji et al. 2009), and interfere with regulatory mechanisms in the airways, such as the release of epithelium‐derived relaxing factor which induces relaxation of airway smooth muscle and submucosal blood vessels (Prazma et al. 1994; Fedan et al. 2004; Wu et al. 2004).

There is a large body of evidence supporting the notion that NGF can alter ion transport in nonpulmonary cells. For example, in PC12 cells, NGF has been demonstrated to increase Na^+^ current (Pollock et al. 1990), increase Na^+^/K^+^‐pump activity (Boonstra et al. 1983), induce type II/IIA Na^+^ channel gene expression (D'Arcangelo et al. 1993), and induce expression of the peripheral nerve‐Na^+^ channel gene, PN42 (Toledo‐Aral et al. 1995). In the renal medullary thick ascending limb (MTAL), NGF inhibits the Na^+^/H^+^ exchanger 1 (NHE1; Watts and Good 2002).

In view of the substantial evidence linking NGF to pulmonary diseases, as well NGF's ability to alter ion transport in PC12 and the MTAL cells, we hypothesized that NGF is involved in the regulation of ion transport in airway epithelial cells. Our results suggest that NGF produces a rapid reduction in amiloride‐sensitive Na^+^ transport in human airway epithelial cells, that is accompanied by phosphorylation of Erk1/2 and the β‐subunit of ENaC.

## Methods

### Cell culture

**Figure 1.**
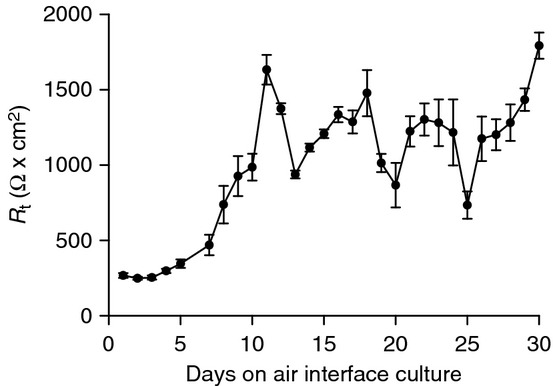
Time course of the development of *R*_t_ in air–liquid interface (ALI)‐cultured normal human bronchial epithelial cells (NHBE). *R*_t_ was monitored over 30 days on ALI. NHBE cells generated and maintained high resistance after 10 days on ALI culture (*n *= 1 donor, 12 replicates).

Normal human bronchial epithelial cells (NHBE, CC‐2540S; Lonza, Walkersville, MD) were cultured according to the manufacturer's instructions. NHBE cells were seeded and expanded (<20 doublings) in a T‐75 flask supported by bronchial air–liquid interface (B‐ALI) growth media containing the recommended supplements (B‐ALI BulletKit, 193514; Lonza). Cells were grown to 80–90% confluence. Following trypsinization (100 *μ*L/cm^2^, 5 min, 25°C), the cells were transferred to semipermeable rat tail collagen (354236; BD Biosciences, San Jose, CA)‐coated polyester transwell inserts (0.4 *μ*m pore size; 0.33 cm^2^; 3470; Corning, Corning, NY) at a density of 50,000 cells/well. The cells were cultured using B‐ALI growth medium in both apical and basolateral compartments for approximately 3 days or until the cells became confluent. Once confluent, the cells were placed under ALI culture conditions, and only supported by B‐ALI differentiation media containing the recommended supplements in the basolateral compartment. The cells were allowed to grow for 21 days under ALI conditions with daily media changes. Growth to confluence was monitored by measuring transepithelial resistance (*R*_t_, EVOM^2^; World Precision Instruments, Sarasota, FL), and cells were used after 21 days of growth and when *R*_t_ was approximately 1000 Ω·cm^2^ (Fig. 1).

### Cell imaging

Differentiation into a ciliated pseudo‐stratified epithelial cell culture was confirmed through a series of imaging and staining techniques. The membrane inserts were fixed in 10% buffered formalin, rinsed in Hank's balanced salt solution (37°C), dehydrated in graded series of ethanol, cleared in xylene, infiltrated, and embedded in paraffin. Sections (5 *μ*m) were placed on microscope slides, and stained with hematoxylin and eosin (H&E). The samples were imaged on an Olympus IX70 photomicroscope (Shinjuku, Tokyo, Japan). H&E staining revealed the presence of a pseudo‐stratified epithelial cell culture.

Mucus production was confirmed using alcian blue staining. Membrane inserts were stained apically with a 1% alcian blue solution (3% acetic acid, pH 2.5) for 30 sec. The alcian blue solution was removed, and cells were imaged on a Zeiss Axiovert 100 microscope (Oberkochen, Germany) equipped with a Pixera Pro 150ES camera (Santa Clara, CA).

The presence of cilia was confirmed by immunofluorescence from β‐tubulin, scanning electron microscopy (SEM), and transmission electron microscopy (TEM). For β‐tubulin immunofluorescence membrane inserts were washed with PBS, fixed with apically applied methanol (4°C), and stained using a monoclonal antitubulin‐FITC antibody (F2043; Sigma‐Aldrich, St. Louis, MO). β‐tubulin was detected using immunofluorescence on a Axiovert 100 microscope equipped with a Pixera Pro 150ES camera. For SEM, the samples were fixed in 4% paraformaldehyde fixative and postfixed in osmium tetroxide. The cells were dehydrated in an ethanol series, dried using hexamethyldisalizane as the final solution and coated with gold/palladium. The samples were imaged on a Hitachi 4800 field emission scanning electron microscope (Chiyoda, Tokyo). For TEM, the samples were fixed in Karnovsky's fixative (2.5% gluteraldehyde, 2.5% paraformaldehyde in 0.1 mol/L sodium cacodylic buffer), postfixed in osmium tetroxide, mordanted in 1% tannic acid, and stained en bloc in 0.5% uranyl acetate. The cells were dehydrated in an ethanol series and embedded in Epon, sectioned, and stained with Reynold's lead citrate and an aqueous uranyl acetate. The sections were imaged on a JEOL 1220 transmission electron microscope (Peabody, MA).

### Ion transport in cultured epithelial cells

Transwell cell culture inserts were placed into Ussing chambers (Physiologic Instruments, San Diego, CA). Cells were bathed in modified Krebs‐Henseleit solution (MKHS, 113.0 mmol/L NaCl; 4.8 mmol/L KCl; 2.5 mmol/L CaCl_2_; 1.2 mmol/L KH_2_PO_4_; 1.2 mmol/L MgSO_4_; 25.0 mmol/L NaHCO_3_; and 5.7 mmol/L glucose; pH 7.4; 37°C; gassed with 95% O_2_, 5% CO_2_) in both apical and basolateral hemi‐chambers. Cells were stabilized under open‐circuit conditions before applying a 0 mV voltage‐clamp using an automatic voltage/current amplifier (EVC 4000, World Precision Instruments or VCC MC8; Physiological Instruments). Short‐circuit current (*I*_sc_) and *R*_t_ were monitored to investigate whether responses to agents were due to a change in transcellular as opposed to paracellular ion transport. This was done by delivering 5‐sec long, 1 mV pulses every 55 sec and calculating *R*_t_ using Ohm's law.

### Effects of NGF and agents on ion transport

After stabilization of *I*_sc_, concentration–response curves for apical and basolateral additions of NGF (SRP3018, 0.001–100 ng/mL NGF; Sigma‐Aldrich) were generated. The ability of NGF to alter ion transport responses to amiloride (3.5 × 10^−5^ mol/L), NPPB (10^−4^ mol/L), and ouabain (10^−4^ mol/L) were evaluated in the absence or presence of 1 ng/mL NGF. To investigate whether the NGF receptor, trkA, mediates the effects of NGF on ion transport, cells were incubated for 30 min with the nonspecific tyrosine kinase inhibitor, K‐252a (10 nmol/L, apical, K1639; Sigma‐Aldrich), or DMSO (0.004%). Subsequently, responses to the ion transport inhibitors were obtained in the absence or presence of NGF. To investigate whether trkA activates the Erk1/2 signaling pathway during responses to NGF, cells were incubated 60 min with the Erk1/2‐specific inhibitor, PD‐98059 (30 *μ*mol/L, apical and basolateral; 9900L, Cell Signaling, Danvers, MA) or DMSO (0.06%). Responses to the ion transport inhibitors mentioned above were obtained in the absence or presence of NGF after 60 min incubation with either PD‐98059 or DMSO. To investigate whether NGF could alter ion transport after a 24‐ and 48‐h incubation, cells were incubated apically with MKHS only or MKHS containing 1 ng/mL of NGF. Responses to known ion transport inhibitors were evaluated to investigate changes in ion transport. To investigate whether the effects of NGF involved changes in Na^+^/K^+^‐ATPase activity, the apical membrane was permeabilized with nystatin (50 *μ*mol/L, N6261; Sigma‐Aldrich) and responses to ouabain generated in the absence or presence of NGF were compared. Results are expressed as a percent change in baseline *I*_sc_. Results obtained in the presence of nystatin are expressed as a percent change from the *I*_sc_ value in the presence of nystatin.

### NGF stability and concentration after prolonged incubation periods

To investigate the stability of NGF in sterile MKHS (the medium in which cells were bathed in Ussing chambers), MKHS with or without 1 ng/mL NGF was added apically to transwell cell culture inserts, lacking or containing cells, and incubated at 37°C for 5 min, 6, 24, and 48 h. The basolateral chamber contained MKHS only. An enzyme linked immunosorbent assay (ELISA; ab99986; Abcam, Cambridge, MA) was used to measure the apical NGF concentration and endogenous release of NGF from epithelial cells. Results were expressed as a percent change from the initial NGF concentration.

### Protein analysis using western blots of NHBE cells incubated with NGF

Cell lysates were prepared from NHBE cells cultured under ALI culture for 21 days. Cells were treated with K‐252a (10 nmol/L, 30 min, apical) or PD‐98059 (30 *μ*mol/L, 60 min, apical and basolateral) prior to incubation with either MKHS or MKHS containing 1 ng/ml NGF. Cells were washed with PBS (4°C) and lysed with Pierce RIPA buffer (89901, Thermo Fisher Scientific, Waltham, MA) containing halt protease inhibitor (78430, Thermo Fisher Scientific), 5 mmol/L EDTA (1960851, Thermo Fisher Scientific), and phosphatase inhibitor cocktail 2 (P5726, Sigma‐Aldrich). Cell lysates were sonicated for two rounds of 10‐sec pulses, centrifuged at 14,000 rpm for 5 min, and protein concentrations were determined using a BCA protein assay (23227, Thermo Fisher Scientific). Samples were denatured in Laemmli sample buffer (161‐0737, BioRad, Hercules, CA) containing β‐mercaptoethanol (M‐6250, Sigma‐Aldrich) at 95°C. Proteins were separated on a 4–15% mini‐protein TGX Gel (456‐1034, BioRad), and transferred to a nitrocellulose membrane (162‐0112, BioRad). Membranes were blocked for 1 h with Odyssey blocking buffer (927‐40000, Li‐Cor, Lincoln, NE) before being probed for 1 h with the primary antibodies for β‐ENaC and phosphorylated‐β‐ENaC (T615)(ab28668 and ab79172, Abcam, Cambridge, MA), Erk 1/2 and phosphorylated‐Erk 1/2 (ab36991 and ab4819, Abcam), and β‐actin (ab8227, Abcam). Membranes were washed three times with TBST‐20 (28360, Thermo Fisher Scientific), incubated with the secondary antibody, IRDye 680LT (926‐68021, Li‐Cor) or IRDye 800CW (926‐32210, Li‐Cor), washed three more times with TBST‐20, and then developed and analyzed on an Odyssey infrared imaging system (9120, Li‐Cor) with software version 3.0.30. Membranes were stripped between incubations with primary antibodies using OneMinute Advanced Western Blot Stripping Buffer (GM6031, GM Biosciences, Rockville, MD). Results are expressed as a percent of control.

### Statistical analysis

Statistical comparisons between groups containing multiple donors were performed with SAS/STAT software (v9.2) for Windows, utilizing the Proc Mixed function to carry out a one‐way analysis of variance. Statistical comparisons between groups involving cells from a single donor were performed with SigmaPlot 11.0 to carry out a one‐way analysis of variance. Differences were considered significant at p < 0.05.

## Results

### Airway epithelial cell characteristics

Cells were cultured under ALI conditions for 30 days, and *R*_t_ was measured daily (Fig. 1). The cells generated high epithelial resistance similar to that reported previously in NHBE cultures grown using ALI conditions (Lin et al. 2007). The cells reached a maximum *R*_t_ on day 30 (1792 ± 87 Ω·cm^2^), and averaged an *R*_t_ of 1204 ± 66 Ω·cm^2^ from day 10 onward.

**Figure 2.**
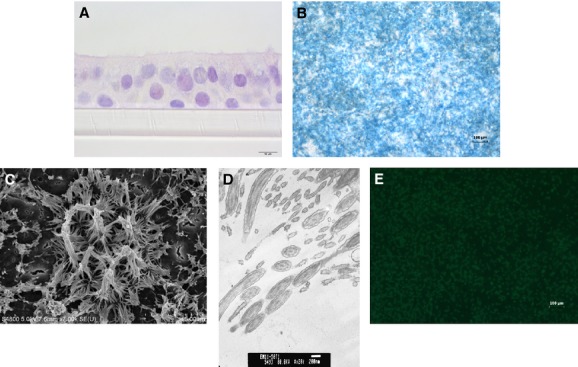
Confirmation of normal human bronchial epithelial cell (NHBE) differentiation. The differentiation of NHBE cells was confirmed through several imaging techniques. (A) Hematoxylin and eosin staining revealed a pseudo‐stratified epithelium with the presence of cilia, (B) alcian blue staining confirmed mucus production, and (C) scanning electron microscopy revealed the presence of cilia on the apical surface. These structures were confirmed to be cilia through the use of (D) transmission electron microscopy, which revealed the presence of a 9 + 2 doublet of microtubules, and (E) immunofluorescence for β‐tubulin.

The NHBE cells grew into a well‐differentiated airway epithelium resembling that of in situ tissue. H&E staining (Fig. 2A) revealed that cells differentiated into a pseudo‐stratified epithelium with the presence of cilia on the apical membrane. The production of mucus was confirmed using alcian blue staining (Fig. 2B). The formation of cilia was confirmed using SEM (Fig. 2C), TEM (Fig. 2D), and immunofluorescence for β‐tubulin, a marker for cilia (Fig. 2E). The TEM imaging revealed the presence of a 9 + 2 doublet in cilia, indicating that the structures were not microvilli.

After 21 days of ALI culture, cells placed into a Ussing chamber and allowed to equilibrate under open‐circuit conditions displayed a *V*_t_ value of −7.1 ± 3.4 mV. Basal *I*_sc_ was 5.9 ± 1.0 *μ*A/cm^2^ (average of 4 donors). Preliminary NGF concentration–response curves (0.001–100 ng/mL) were generated to investigate whether NGF could elicit bioelectrical responses in airway epithelial cells (data not shown). The addition of NGF to the apical membrane resulted in a reduction in *I*_sc_. NGF did not evoke responses at any concentration after being applied basolaterally. The concentrations used to generate these NGF concentration–response curves are clinically relevant and reflect levels found in both the blood and BAL‐fluid samples of infants experiencing respiratory infections and after surgery (Tortorolo et al. 2005). Based on the concentration–response analysis, 1 ng/mL of NGF applied to the apical membrane was used routinely for the remaining experiments as this concentration provided the maximum response.

### NGF reduces amiloride‐sensitive Na^+^ transport

**Figure 3.**
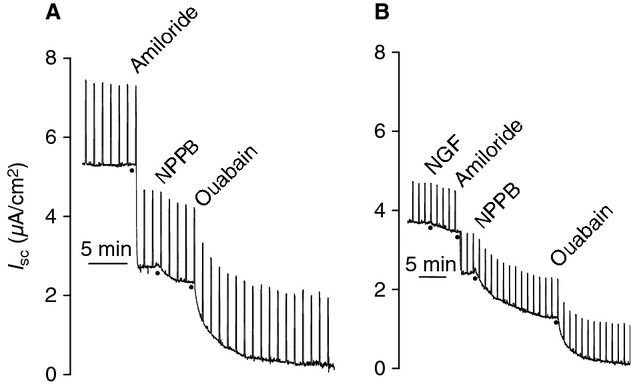
Representative *I*_sc_ tracings of responses to known ion transport inhibitors. Once *I*_sc_ stabilized, responses to amiloride (3.5 × 10^−5^ mol/L), NPPB (10^−4^ mol/L), and ouabain (10^−4^ mol/L) were generated in the (A) absence or (B) presence of 1 ng/mL nerve growth factor (NGF).

**Figure 4.**
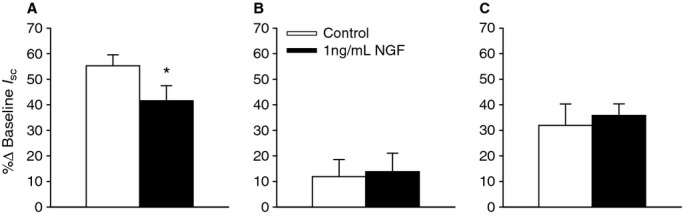
Effect of nerve growth factor (NGF) on ion transport. Responses to (A) amiloride, (B) NPPB, and (C) ouabain were calculated as a percent change from baseline (4 donors). NGF significantly reduced the amiloride response (*P* = 0.0127).

**Figure 5.**
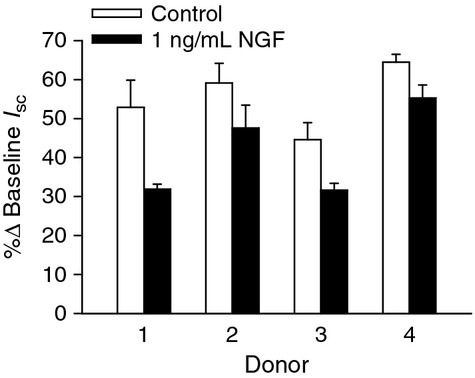
Effect of nerve growth factor (NGF) on amiloride‐sensitive Na^+^ transport. Normal human bronchial epithelial cells cultured from four donors demonstrated consistent reductions in Na^+^ transport in the presence of 1 ng/mL NGF.

To investigate the basis of the bioelectric responses to NGF, ion transport inhibitors were added apically or basolaterally as appropriate, in the absence (Fig. 3A) or presence (Fig. 3B) of 1 ng/mL NGF. The addition of 1 ng/mL NGF to the apical chamber resulted in a 5.3 ± 1.5% reduction in *I*_sc_. In the absence of NGF, amiloride (3.5 × 10^−5^ mol/L, apical), which inhibits the ENaC, caused a 55.3 ± 4.6% reduction in *I*_sc_, but in the presence of NGF, amiloride reduced the *I*_sc_ by 41.6 ± 3.0% (Fig. 4A; *P* = 0.0127). NGF had no effect on the response to the Cl^−^ channel inhibitor, NPPB (Fig. 4B; apical, 10^−4^ mol/L; control 11.9 ± 1.6%, NGF 13.8 ± 1.7%; *P* = 0.800) or the Na^+^/K^+^‐ATPase inhibitor, ouabain (Fig. 4C; basolateral; control 31.9 ± 2.6%, NGF 35.8 ± 2.6%; *P* = 1.00). The decrease in amiloride‐sensitive Na^+^ transport by NGF was observed in the epithelium from four different donors (Fig. 5). Because of the consistency of the amiloride response between donors, it was decided to use the epithelial cells from one donor for the remaining experiments.

**Figure 6.**
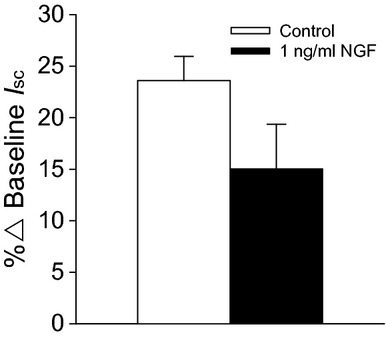
Effect of nerve growth factor (NGF) on responses to apically applied amiloride after 30 min. Although the response to amiloride was reduced in the presence of NGF, this reduction was not significant. *n* = 4.

In the above experiments the cells had been incubated with NGF until a stable response was obtained, that is, ~5 min, before amiloride was applied. To investigate whether the effect of NGF on Na^+^ transport was maintained for longer period of time, responses to amiloride were generated following 30‐min incubation with 1 ng/mL NGF. Under these conditions responses to amiloride were reduced in magnitude in the presence of NGF, albeit not significantly (Fig. 6).

### NGF reduces amiloride‐sensitive Na^+^ transport through a trkA receptor‐mediated pathway

**Figure 7.**
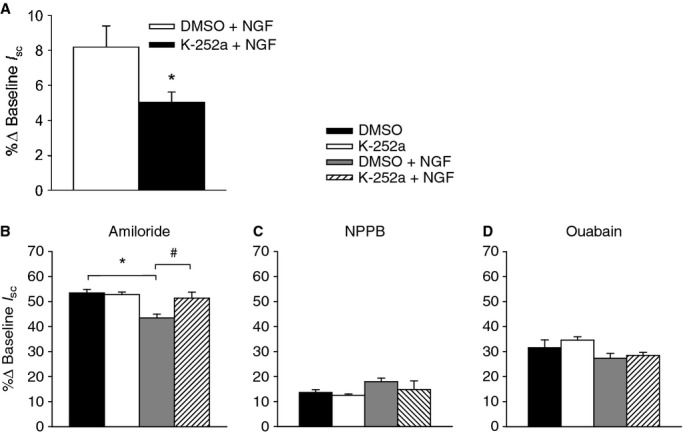
Effect of nerve growth factor (NGF)‐induced trkA activation on amiloride‐sensitive Na^+^ transport. Cells were either incubated apically with the nonspecific tyrosine kinase inhibitor, K‐252a, or DMSO for 30 min prior to generating responses to (A) NGF, (B) amiloride, (C) NPPB, and (D) ouabain in the absence or presence of NGF. Incubation with K‐252a significantly attenuated both the (A) NGF response (*P* = 0.04) and (B) the NGF induced reduction in amiloride‐sensitive Na^+^ (*P = 0.002; ^#^*P* = 0.041). DMSO *n* = 4; all other groups *n* = 6.

To investigate whether the trkA receptor, which has been identified histologically on the apical surface of airway epithelial cells (Wu et al. 2006), mediates the reduction in amiloride‐sensitive Na^+^ transport in response to NGF, cells were incubated for 30 min with the nonspecific tyrosine kinase inhibitor K‐252a (10 nmol/L; apical) or DMSO as the vehicle control (0.004%). K‐252a significantly reduced NGF responses (Fig. 7A). K‐252a itself did not have an effect on amiloride‐sensitive *I*_sc_, but K‐252a attenuated the NGF‐induced reduction in Na^+^ transport (Fig. 7B). There were no significant changes in the responses to NPPB or ouabain, indicating that there were no changes in Cl^−^ transport or Na^+^/K^+^‐ATPase activity (Fig. 7C and D). These results suggest that the trkA receptor mediates bioelectric responses to NGF and its activation inhibits amiloride‐sensitive Na^+^ transport.

### NGF reduces amiloride‐sensitive Na^+^ transport through a trkA/Erk1/2‐mediated pathway

**Figure 8.**
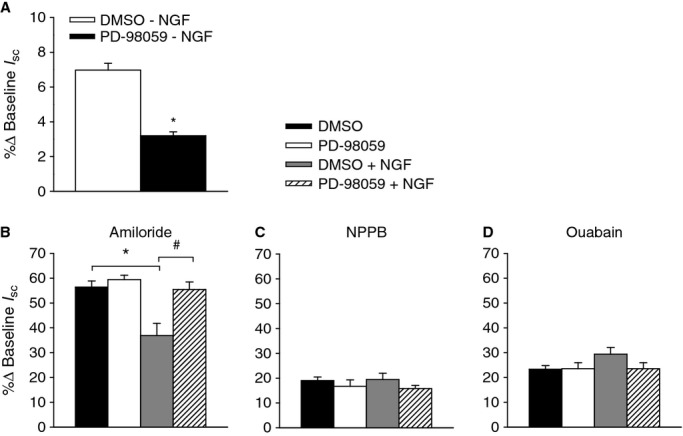
The involvement of the trkA downstream signaling pathway, Erk 1/2, in the nerve growth factor (NGF)‐induced reduction in amiloride‐sensitive Na^+^ transport. Cells were either incubated apically and basolaterally with the specific Erk 1/2 inhibitor, PD‐98059, or DMSO for 30 min prior to generating responses to (A) NGF, (B) amiloride, (C) NPPB, and (D) ouabain in the absence or presence of NGF. Incubation with PD‐98059 significantly attenuated both the (A) NGF response (*P* = 0.001) and (B) the NGF induced reduction in amiloride‐sensitive Na^+^ transport (*P = 0.002; ^#^*P *= 0.012). DMSO *n* = 4; all other groups *n* = 6.

The trkA receptor, when activated by NGF, can activate several intracellular signaling cascades, including the Erk1/2 signaling pathway (Segal and Greenberg 1996). To investigate whether the NGF‐induced reduction in amiloride‐sensitive Na^+^ transport involves the activation of the Erk1/2 signaling cascade, cells were incubated with either the Erk1/2 inhibitor, PD‐98059 (30 *μ*mol/L; apical and basolateral), or DMSO (0.06%) 60 min prior to generating responses to ion transport inhibitors in the absence or presence of NGF. PD‐98059 itself significantly reduced the response to NGF (Fig. 8A), and also attenuated the NGF‐induced reduction in amiloride‐sensitive Na^+^ transport (Fig. 8B). Again, there were no significant changes in the responses to NPPB or ouabain, indicating that there were no changes in Cl^−^ transport or Na^+^/K^+^‐ATPase activity (Fig. 8C and D). These findings suggest that Erk1/2 participates in the reduction in amiloride‐sensitive Na^+^ transport in response to NGF.

### NGF does not affect Na^+^/K^+^ ATPase activity

**Figure 9.**
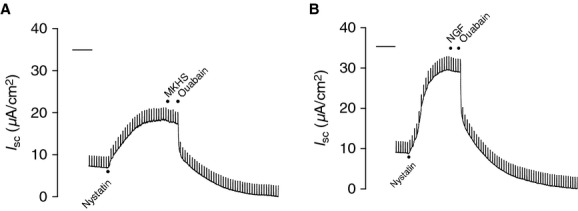
The effect of nerve growth factor (NGF) in epithelial cells permeabilized apically with nystatin. Cells were placed into Ussing chambers and allowed to equilibrate prior to adding nystatin (50 *μ*mol/L) to the apical chamber. Nystatin caused a large increase in *I*_sc_. Responses to ouabain were generated in the (A) absence (vehicle control – modified Krebs‐Henseleit solution [MKHS]) or (B) presence of 1 ng/mL NGF. NGF did not elicit bioelectric responses when applied apically to the permeabilized cells, and did not alter Na^+^/K^+^‐ATPase activity as there were no differences in the response to ouabain. Control *n* = 6, NGF *n* = 4. Scale bar = 10 min.

Although apically applied NGF did not result in significant changes in Na^+^/K^+^‐ATPase activity (Fig. 4C), Erk 1/2 activation has been reported to affect Na^+^/K^+^‐ATPase expression (Guerrero et al. 2001) and activity (Lei et al. 2008) in alveolar epithelial cells. It is possible, therefore, that NGF could affect Na^+^/K^+^‐ATPase activity in addition to Na^+^ transport. To investigate whether NGF affects Na^+^/K^+^‐ATPase activity, the apical membrane was permeabilized with nystatin (50 *μ*mol/L; apical), and responses to ouabain were obtained in the absence (Fig. 9A) or presence (Fig. 9B) of NGF (1 ng/mL). In the presence of nystatin, apically applied NGF had no effect on *I*_sc_, in contrast to responses of cells in the absence of nystatin (data not shown). There was no significant difference in the responses to ouabain between the vehicle control and NGF‐treated groups (Control 98.4 ± 1.2%, NGF 99.7 ± 0.8%; data not shown). These results suggest that the NGF‐induced Erk 1/2 activation is a localized signaling event which occurs at the apical membrane that affects amiloride‐sensitive Na^+^ transport but not basolateral Na^+^/K^+^‐ATPase activity.

### Prolonged incubation with NGF

**Figure 10.**
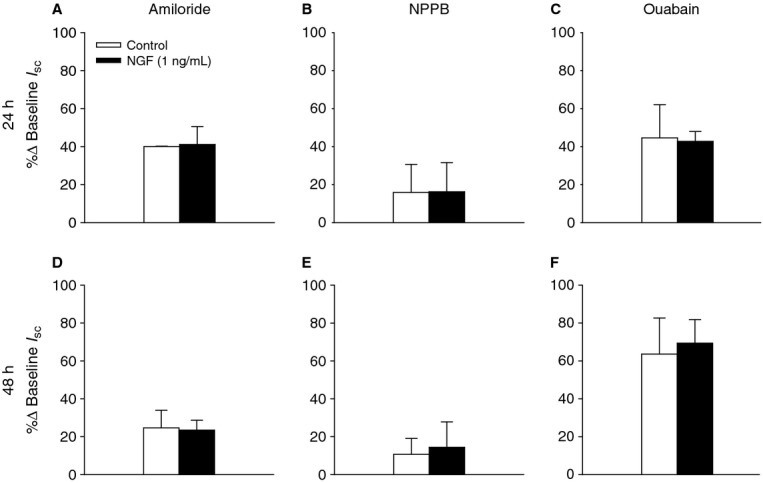
Effect of prolonged incubation with nerve growth factor (NGF) on ion transport. Responses to known ion transport inhibitors were generated after incubating cells 24 h (A–C) and 48 h (D–F) with 1 ng/mL NGF. There were no difference in response to amiloride (A and D), NPPB (B and E), and ouabain (C and F) between control or NGF treated cells following either a 24‐ or 48‐h incubation (2 donors).

To examine the effect of prolonged incubation with NGF, cells which were incubated for 24 h (Fig. 10A–C) or 48 h (Fig. 10D–F) with apically applied NGF (1 ng/mL) or MKHS, were placed into the Ussing system to measure responses to amiloride. NGF did not reduce the amiloride‐sensitive Na^+^ transport in cells incubated for either 24 or 48 h, which suggested at first that the reduction in Na^+^ transport is a transient, nongenomic cellular response to NGF.

We considered the possibility that the lack of a lasting effect of NGF on amiloride‐sensitive Na^+^ transport could be due to a reduction in NGF concentration during the incubation period or due to desensitization of the cells. Therefore, an ELISA specific for β‐NGF was used to measure the NGF concentration in the apical solution. Transwell inserts with and without cells were incubated for 5 min, or 6, 24, and 48 h apically with either NGF (1 ng/mL) or MKHS. After incubation, the apical solution was collected and analyzed for NGF. There were no detectable levels of endogenous NGF in any of the transwell inserts with or without cells incubated with MKHS. However, following 5‐min incubation on inserts which contained cells, the NGF concentration was reduced by 93%. Transwell inserts which did not contain cells and were incubated with NGF did not reveal a reduction in NGF concentration, indicating that NGF was either metabolized by epithelial proteases or NGF was internalized into the cells with the trkA receptor, as reported previously (Saragovi et al. 1998).

In the presence of epithelial cells, 94.7 ± 0.4%, 94.9 ± 0.5%, and 96.1 ± 0.1% reductions in NGF concentration were observed following 6, 24, and 48 h of incubation, respectively. In inserts lacking cells, which did not reveal a decrease in NGF after 5 min, there were 84.2 ± 2.3%, 99.8 ± 0.1%, and 99.9 ± 0.0% reductions in NGF concentration following a 6‐, 24‐, and 48‐h incubation, respectively. The reduction of NGF in transwell inserts without cells demonstrates that NGF is degraded when incubated at 37°C in MKHS for a prolonged period of time.

### NGF mediates ENaC phosphorylation through a trkA‐Erk 1/2 signaling pathway

**Figure 11.**
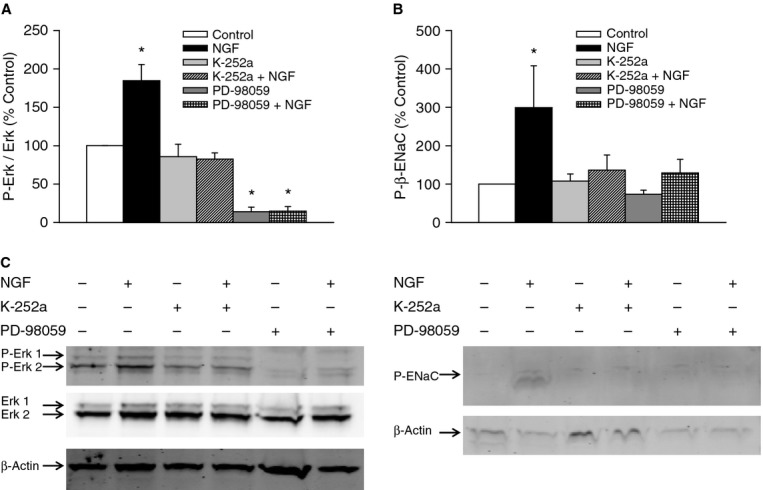
Western blots showing the effects of nerve growth factor (NGF) on Erk 1/2 activation and ENaC phosphorylation. Cells were incubated apically with modified Krebs‐Henseleit solution (MKHS; control) or 1 ng/ml NGF in MKHS for 5 min. (A) NGF activated the Erk 1/2 signaling pathway. This activation was inhibited by K‐252a and PD‐98059. (B) NGF‐mediated activation of Erk 1/2 resulted in ENaC phosphorylation, and was inhibited with K‐252a and PD‐98059. (C) Representative blots for Erk 1/2 (42 and 44 kDa), phosphorylated Erk 1/2 (P‐Erk; 44 and 45 kDa), phosphorylated ENaC (76 kDa), and the loading control, β‐actin (47 kDa). *n* = 4. *P < 0.05.

**Figure 12.**
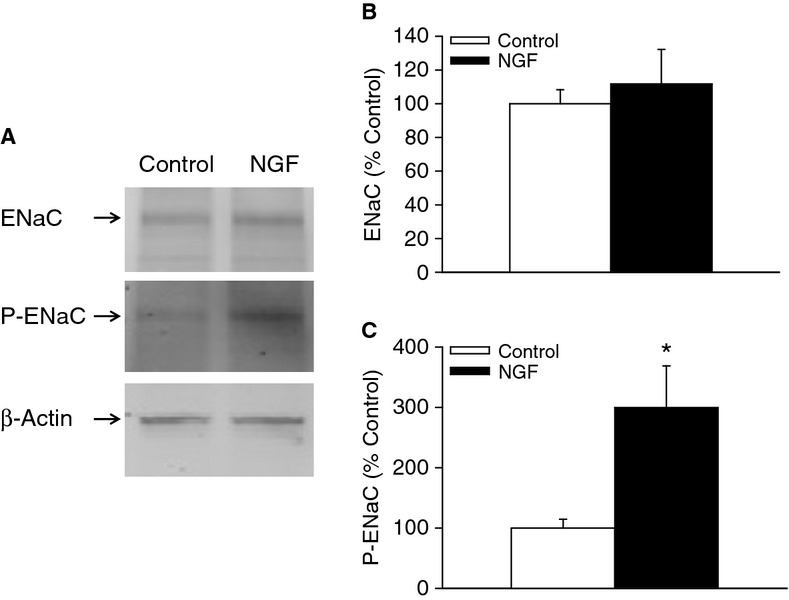
Western blots showing the effect of nerve growth factor (NGF) on β‐ENaC. (A and C) Cells incubated with NGF for 5 min demonstrated a threefold increase in phosphorylated‐β‐ENaC (76 kDa). (B) Blots were stripped and probed for β‐ENaC (75 kDa) and β‐Actin (42 kDa). NGF did not affect β‐ENaC levels. *n* = 6, *P < 0.05.

In the Ussing system experiments, we demonstrated that NHBE cells respond electrophysiologically to apically applied NGF, and, in the presence of NGF, amiloride‐sensitive Na^+^ transport was attenuated. Furthermore, K‐252a and PD‐98059 inhibited NGF's effect. Apically permeabilized epithelial cells were unresponsive to NGF, and NGF was found to have no effect on the Na^+^/K^+^‐ATPase activity. Epidermal growth factor (EGF) also has been shown to activate an Erk 1/2‐mediated pathway and selectively inhibit ENaC without affecting Na^+^/K^+^‐ATPase activity (Shen and Cotton 2003). Thus, the ability of NGF to activate the Erk 1/2 signaling, as well as the possibility that NGF, acting through this second messenger pathway, could phosphorylate ENaC was investigated. Treatment with apical NGF (1 ng/mL, 5 min) resulted in a twofold Erk 1/2 activation (Fig. 11A) and a threefold increase in β‐ENaC phosphorylation (Fig. 11B) as compared to controls. The increases in Erk 1/2 activity and β‐ENaC phosphorylation were blocked by K‐252a (10 nmol/L, apical) and PD‐98059 (30 *μ*mol/L, apical and basolateral; Fig. 11C). Although there was a threefold increase in β‐ENaC phosphorylation, there were no changes in total β‐ENaC (Fig. 12). These results could suggest that NGF, acting though a trkA‐Erk 1/2‐mediated pathway, causes the phosphorylation of ENaC, which could be associated with reduced Na^+^ transport.

## Discussion

The addition of NGF to the apical membrane of NHBE cells decreased *I*_sc_, and reduced amiloride‐sensitive Na^+^ transport. NGF did not affect Cl^−^ transport or Na^+^/K^+^‐ATPase activity, as there were no effects on the responses to NPPB or ouabain, and NGF was ineffective in the presence of nystatin to short circuit the apical membrane. The nonspecific tyrosine kinase inhibitor, K‐252a, as well as the specific Erk 1/2 inhibitor, PD‐98059, attenuated both the response to NGF as well as the NGF‐induced reduction in amiloride‐sensitive Na^+^ transport. NGF did not reduce amiloride‐sensitive Na^+^ transport after a 24‐ or 48‐h incubation, which appears to be a result of NGF degradation. This finding also implies that NGF did not elicit early genomic effects to change ion transporter expression under the conditions of our experiments. The results would suggest that NGF, acting through a trkA‐Erk1/2‐ mediated signaling pathway leading to ENaC phosphorylation, reduces Na^+^ transport in airway epithelial cells.

There has been extensive work to investigate the regulatory mechanisms controlling ENaC, as ENaC not only plays a critical role in airway fluid clearance but also in the kidney where it is involved in maintaining blood volume and pressure (Bhalla and Hallows 2008). The regulation of ENaC can be controlled through a variety of both extrinsic and intrinsic factors, both through genomic effects, such as protein synthesis, and nongenomic effects, such as the change in the number of ENaC channels expressed on the membrane or a change in ENaC kinetics. The rapidity of the NGF‐induced reduction in Na^+^ transport would suggest that the mechanism involves a nongenomic mechanism(s).

The addition of NGF to the apical membrane, but not the basolateral membrane, resulted in a decrease in *I*_sc_. Previous work suggests the specific trafficking of the trkA receptor to the apical membrane and the p75 receptor to the basolateral membrane (Wu et al. 2006). The lack of bioelectric response when NGF was applied to the basolateral membrane, as well as the significant reduction in the response to apically applied NGF in the presence of K‐252a, would suggest the interaction of NGF with the apical trkA receptor led to altered ion transport.

NGF, specifically protein loops 2 and 4, interacts with the trkA receptor, but not with the p75 receptor, to phosphorylate and activate the Erk1/2 signaling pathway (Xie et al. 2000). The interaction between NGF and trkA occurs within 1 min, with maximum trkA activation occurring after a 5‐min incubation with NGF (Kaplan et al. 1991). Activated Erk 1/2 mediates the phosphorylation of two specific threonine residues on the β‐ and γ‐ENaC subunits located near a PXTP motif on the cytosolic C‐terminus (Shi et al. 2002). The phosphorylation of βThr‐613 and γThr‐623 on ENaC results in much higher binding affinity between the WW domain on the E3 ubiquitin‐protein ligase Nedd4 and the PXTP motif on ENaC, resulting in the ubiquitination and downregulation of ENaC. Although NGF activates the Erk1/2 signaling pathway downstream of the trkA receptor and activated Erk1/2 has been shown to phosphorylate and downregulate ENaC, no previous study has investigated NGF's ability to activate the Erk1/2 signaling pathway and the resulting phosphorylation of ENaC in airway epithelial cells.

The ELISA data demonstrated a rapid reduction (93% in 5 min) in apically applied NGF in the presence of epithelial cells. Without NGF present in the apical bath continued signal activation would not be expected to occur. However, NGF has been shown to cause trkA activation in 1 min (Kaplan et al. 1991) and maximum Erk activation 1.5 min after exposure (Saragovi et al. 1998). This rapid signaling coincides with the rapid electrophysiological responses stimulated by NGF. Thus, the bioelectric and the biochemical events would appear to have followed a similar time course. After 30‐min incubation with NGF, at a time when NGF concentration in the chamber had diminished substantially, the effect of amiloride was blunted and was no longer significant. This must reflect the decline in NGF levels during this period. But the fact that there was a trend toward an effect on responses to amiloride could suggest, interestingly, that NGF had initiated a longer term, “hit and run” effect during the first 5 min of incubation.

Although we observed a threefold increase in β‐ENaC phosphorylation after a 5‐min incubation with NGF, we did not observe a reduction in total β‐ENaC (Fig. 12) over the measurement period. Falin and Cotton (2007) using MDCK cells demonstrated that EGF induced an Erk‐mediated reduction in Na^+^ transport as a result of ENaC phosphorylation, but a reduction in ENaC surface expression was not observed until after 60 min. It is possible that longer incubations with NGF (under conditions in which NGF levels might somehow be stabilized in MKHS) could reveal ENaC degradation late in the response. During diseases such RSV infections, NGF is significantly increased in the cell fraction of the bronchoalveolar lavage fluid, but not in the serum (Tortorolo et al. 2005). This increased NGF level has been attributed to increased production by the infiltrating inflammatory cells. The increased production and release of NGF by inflammatory cells would result in a continuous release of NGF and exposure of the epithelium, and, perhaps, initiate changes in ion transport by mechanisms uncovered during this investigation.

## Acknowledgments

The findings and conclusions in this report are those of the authors and do not necessarily represent the views of the National Institute for Occupational Safety and Health. Mention of brand name does not constitute product endorsement.

## Conflict of Interest

None declared.
